# Archform Comparisons between Skeletal Class II and III Malocclusions

**DOI:** 10.1371/journal.pone.0100655

**Published:** 2014-06-27

**Authors:** Wei Zou, JiaQi Wu, JiuHui Jiang, TianMin Xu, CuiYing Li

**Affiliations:** 1 Department of Orthodontics, Peking University School and Hospital of Stomatology, Beijing, China; 2 Department of Oral Pathology, Peking University School and Hospital of Stomatology, Beijing, China; Université de Poitiers, France

## Abstract

The purpose of this cross-sectional research was to explore the relationship of the mandibular dental and basal bone archforms between severe Skeletal Class II (SC2) and Skeletal Class III (SC3) malocclusions. We also compared intercanine and intermolar widths in these two malocclusion types. Thirty-three virtual pretreatment mandibular models (Skeletal Class III group) and Thirty-five Skeletal Class II group pretreatment models were created with a laser scanning system. FA (the midpoint of the facial axis of the clinical crown)and WALA points (the most prominent point on the soft-tissue ridge)were employed to produce dental and basal bone archforms, respectively. Gained scatter diagrams of the samples were processed by nonlinear regression analysis via SPSS 17.0. The mandibular dental and basal bone intercanine and intermolar widths were significantly greater in the Skeletal Class III group compared to the Skeletal Class II group. In both groups, a moderate correlation existed between dental and basal bone arch widths in the canine region, and a high correlation existed between dental and basal bone arch widths in the molar region. The coefficient of correlation of the Skeletal Class III group was greater than the Skeletal Class II group. Fourth degree, even order power functions were used as best-fit functions to fit the scatter plots. The radius of curvature was larger in Skeletal Class III malocclusions compared to Skeletal Class II malocclusions (*r_WALA3_*>*r_WALA2_*>*r_FA3_*>*r_FA2_*). In conclusion, mandibular dental and basal intercanine and intermolar widths were significantly different between the two groups. Compared with Skeletal Class II subjects, the mandibular archform was more flat for Skeletal Class III subjects.

## Introduction

Archforms play an important role in orthodontic diagnoses and treatment plans. Limitations for tooth movement, especially for incisor retractions, arise from the basal bone where teeth are rooted [1]. If teeth move excessively over alveolar bone, periodontal complications and unstable treatment effects, even the tooth exfoliation may occur [2]. Most orthodontists have realized that the expansion of the dental arch is affected by the shape of the basal bone. Most common complications of orthodontic treatment, such as the relapse,may have much to do with the shape of basal bone. Interestingly, how to define basal bone is not unanimous: Lundstrom put forth the apical base theory to explain basal bone boundaries located at the apical root level [3]. He proposed that the basal bone were not changed by orthodontic tooth movement and that their expansion was limited by the apical base bone. In contrast, Howes defined the basal bone as the narrowest region of the alveolar bone, 8 mm below the gingival [4]. In 2000, Andrews reported “six elements to normal occlusion” theory, Indicating that FA points(the most prominent part of the center of the clinical crown where an orthodontic bracket would be placed in an appliance system)represent the dental arch form and the WALA ridge reflects the basal bone shape at the same vertical level (WALA points: the most prominent point on the soft-tissue ridge at the mucogingival junction; WALA ridge: the band of keratinized soft tissue directly adjacent to the mucogingival line)[5]. It is difficult to locate the exact position of root apex, which used to be as a measurement of supporting bone. Thus, the WALA ridge is easy to identify and might be more clinically reliable than estimation of the root apex. In 2010, Ball used to evaluate the correlation of dental and basal bone of normal occlusion and Class II malocclusion [6]. They concluded that the WALA ridge estimates the basal bone archform. In other several studies[7], [8], the WALA points also have been used to refer the basal bone arch form,proven reliable to represent the basal bone archform.

Dental and basal bone archforms for normal occlusions have been studied as have those for Class I and Class II malocclusions [9]–[11]. However, the association between dental and basal bone arches for Skeletal Class III counterparts is unclear. Thus, we explored differences between Skeletal Class III and II malocclusions in natural pretreatment models to better understand relationships between natural dental and basal bone archforms of skeletal Class III and Class II patients. This research might be interesting and can provide reference for our clinicians in the choice of orthodontic wires of skeletal Class II and III patients.

## Methods

As the pretreatment tooth position or archform can influence the clinical treatment plan, we use the pretreatment casts in this study. The samples were comprised of 68 mandibular pretreatment casts obtained from subjects with 35 skeletal Class II (SC2) and 33 skeletal Class III (SC3) malocclusions. There are 50 females and 18 males (mean age = 20.27 years), of Mongolian ancestry, referred to the Department of Orthodontics, Peking University, School and Hospital of Stomatology. In skeletal Class II group, there are 29 female (mean age = 21.66 years) and 6 male (mean age = 20.17 years). In skeletal Class III group, there are 21 female (mean age = 20.00 years) and 12 male (mean age = 20.75 years).

### Inclusion criterions were as follows

- All subjects are pretreatment models for included samples (no distortions; clear teeth surface).
- The treatment plans are confirmed as combined orthognathic and orthodontic treatments.
- Minor arch length discrepancy for the lower dental arch (crowding ≤2 mm,spacing ≤2 mm).
- For skeletal Class II group (SC2): A canine and molar Class II relationship and ANB angle >5.
- For skeletal Class III group (SC3): A canine and molar Class III relationship and ANB angle <−5.

### Exclusion criterions were as follows

- Dental crowding or space >2 mm.
- Missing or decayed teeth, prosthetic crown.
- Gingival defects or unidentifiable mucogingival junction on the cast.

The study protocol was approved by the Ethical Committee of Peking University Health Science Center and all patients have signed consent forms.

The 68 dental pretreatment cast samples were laser scanned with a computer-assisted 3D scanning system at a resolution of 0.02 mm (R700 laser scanner, 3 shapes, Denmark). Acquired 3D data were analyzed by Rapidform 2006 (INUS Technology, Seoul, Korea).

As previous studies indicated, the FA point [12] is defined as the midpoint of the facial axis of the clinical crown. However, for the first molar, the FA is located at the most prominent point near in line with the mesiobuccal groove. The WALA point [5] is identified as the most prominent point on the soft-tissue ridge directly below the FA of each tooth, perpendicular to the occlusal plane (Figure S1).

FA points (N = 12) and WALA points (N = 12) from the right first molar to the left first molar on each virtual model were used to create the reference-plane with the least squares method. After building the reference plane, subsequent projection of these points (including all FA and WALA points) were expressed on the reference-plane.

The FA points of the two lower center incisors were connected to formulate an imaginary line whose midpoint was defined as FA1. WALA1 was similarly created. Then, point FW1 was generated by bisecting the vector that connected points FA1 and WALA1 and point FW1 was projected into the reference-plane, creating a projection point (FW1_ref), which became the original point to the coordinate (0, 0, 0). FA6, WALA6, FW6 and FW6_ref were created the same way. Thus, the 3D coordinates were built by these three points: FW1_ref, FW6_ref, and FW6 (Figure S2). Then, FA and WALA point coordinates were exported into Microsoft Excel 2010 and distances between the FA and WALA points for each tooth were calculated later with Rapidform 2006.

All point digitizing and measurements were completed by two graduate students in orthodontics who have the requisite experience to conduct these experiments. Inter-operator reliability was examined between two operators. To evaluate intra-operator reliability of FA and WALA point identification, all selected models were re-digitized 2 weeks later by the same operators. The intraclass correlation coefficients (ICC>0.8) indicated high reliability in both of the operators for point locations and archform measurements [13]. In order to more precisely assess the location of the WALA ridge, Data averages from two examiners were used as final measurement outcomes in the data processing and statistical analysis.

The coordinates of projection points (FA_ref and WALA_ref) were exported into Excel 2010, and the distances between FA_ref and WALA_ref were calculated using the formula of the distance between two points (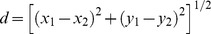) for each tooth. For each model, the respective distances between FA and WALA points for bilateral canines and molars were measured and recorded as Distance FA3, Distance WALA3, Distance FA6, and Distance WALA6.

The data were imported into SPSS 17.0 and subjected to the Kolmogorov-Smirnov test to analyze normality [14]. Data from intercanine and intermolar widths for SC2 group were abnormal distributions, and their dental and basal bone widths in the canine and molar region were compared by using the rank-sum test. Otherwise, data were evaluated with the independent samples t-test [14]. Correlation statistics were used to assess the correlation between the FA and WALA point distances at the canine and molar areas. Pearson correlation coefficients between the intercanine and intermolar widths at the FA and WALA points were also calculated and compared between 2 groups [14]. Scatter diagrams (created by a subsequent projection points on the reference plane) for samples were processed by nonlinear regression analysis via SPSS 17.0.

## Results

### Comparisons of average relative distances between corresponding FA and WALA projection points

The distances between FA_ref and WALA_ref points for each tooth are shown in Table 1 (if the WALA point is located buccally, the value is positive, otherwise the value is negative). A negative distance indicates an anterior area, and a positive value indicates a posterior area in SC2 group. However, positive values are used for both areas in SC3 group (Figure S3).

**Table 1:** Average distance (mm) of WALA points relative to corresponding FA points and their standard deviations in two groups.

TEETH	Skeletal Class II a (n1 = 35)	Skeletal Class III b (n2 = 33)	
	**Average (mm)**	**SD**	**Average (mm)**	**SD**
**46**	2.56	1.26	3	0.91
**45**	1.69	0.95	2.55	1.01
**44**	0.6	1.34	2.16	0.91
**43**	−0.24	1.66	2.42	1.16
**42**	−1.18	1.86	2.51	1.47
**41**	−1.68	1.68	2.24	1.2
**31**	−1.83	1.8	2.32	1.3
**32**	−1.21	1.74	2.49	1.34
**33**	−0.5	1.81	2.34	1.16
**34**	0.58	1.43	2.34	1.09
**35**	1.64	1.08	2.83	1
**36**	2.56	0.57	3.2	0.92

a In skeletal Class II group: a negative distance in anterior area and a positive value in posterior area.

b In skeletal Class III group:positive values in both areas.

### Analysis of intercanine and intermolar widths for FA and WALA points

Descriptive statics for dental and basal bone widths for the two groups are displayed in Table 2. The mandibular intercanine and intermolar widths of FA and WALA points are significantly larger in the SC3 group compared with the SC2 group (P<0.05). Ratios of FA distances (Distance FA3/Distance FA6) for SC3 malocclusions were larger in the SC2 group. Moreover, Table 3 shows that the differences between dental canine width and basal canine width for skeletal Class II malocclusion were not significant statistically,while significant differences were presented in other groups.

**Table 2:** Descriptive statistics for FA and WALA points for canine and molar widths in two groups.

WIDTHS	Class II (n1 = 35)		Class III (n2 = 33)	
	Average (mm)	SD	Average (mm)	SD
**FA6**	52.04	3.09	54.05	3.07
**FA3**	29.18	1.45	30.49	1.54
**WALA6**	56.93	2.85	59.3	2.04
**WALA3**	29.4	2.23	32.03	1.85
**FA ratio**	0.56	0.03	0.57	0.04
**WALA ratio**	0.52	0.04	0.54	0.03

**Table 3:** The results of comparing the dental and basal widths.

Class III Measurements(n = 33)^a^	The comparison of dental lower intercanine width and basal lower intercanine width	The comparison of dental lower intermolar width and basal lower intermolar width	Class II Measurements(n = 35)^b^	The comparison of dental lower intercanine width and basal lower intercanine width	The comparison of dental lower intermolar width and basal lower intermolar width
***t***	−5.842	−20.378	***Z***	−0.704	−5.143
***P***	<0.01	<0.01	***P***	0.481	<0.01

a The results of the independent samples' t test comparing the dental and basal widths for Skeletal Class III malocclusion;

b The results of rank sum test comparing the dental and basal widths for Skeletal Class II malocclusion

Distances between FA and WALA points were highly correlated in the SC3 group for both canine(SC2:correlation coefficient:0.534, P<0.001;SC3:correlation coefficient:0.614, P<0.001)and molar areas(SC2:correlation coefficient:0.873, P<0.001;SC3:correlation coefficient:0.91, P<0.001). Additionally, in both groups, the molar region was highly correlated with respect to distances between molar FA and WALA points (P<0.001). Table 4 shows the correlation analysis between FA and WALA points for both groups.

**Table 4:** The relationship between FA and WALA points at intercanine and intermolar width, corresponding ratio in two groups.

Correlation	Class II (n1 = 35)			Class III (n2 = 33)		
	***Correlation coefficient***	***T value***	***P***	***Correlation coefficient***	***T value***	***P***
**FA6-WALA6**	0.873	10.268	<0.01	0.91	12.241	<0.01
**FA3-WALA3**	0.534	3.627	<0.01	0.614	4.332	<0.01
**FA-WALA ratio (3**–**3/6**–**6)**	0.735	6.22	<0.01	0.637	4.6	<0.01

### Curve fitting of FA and WALA's projection points

We connected FA and WALA projection points through linear interpolation in sequence to acquire superimposed curves for patients in both groups (Figure S4).

**Figure 1:**
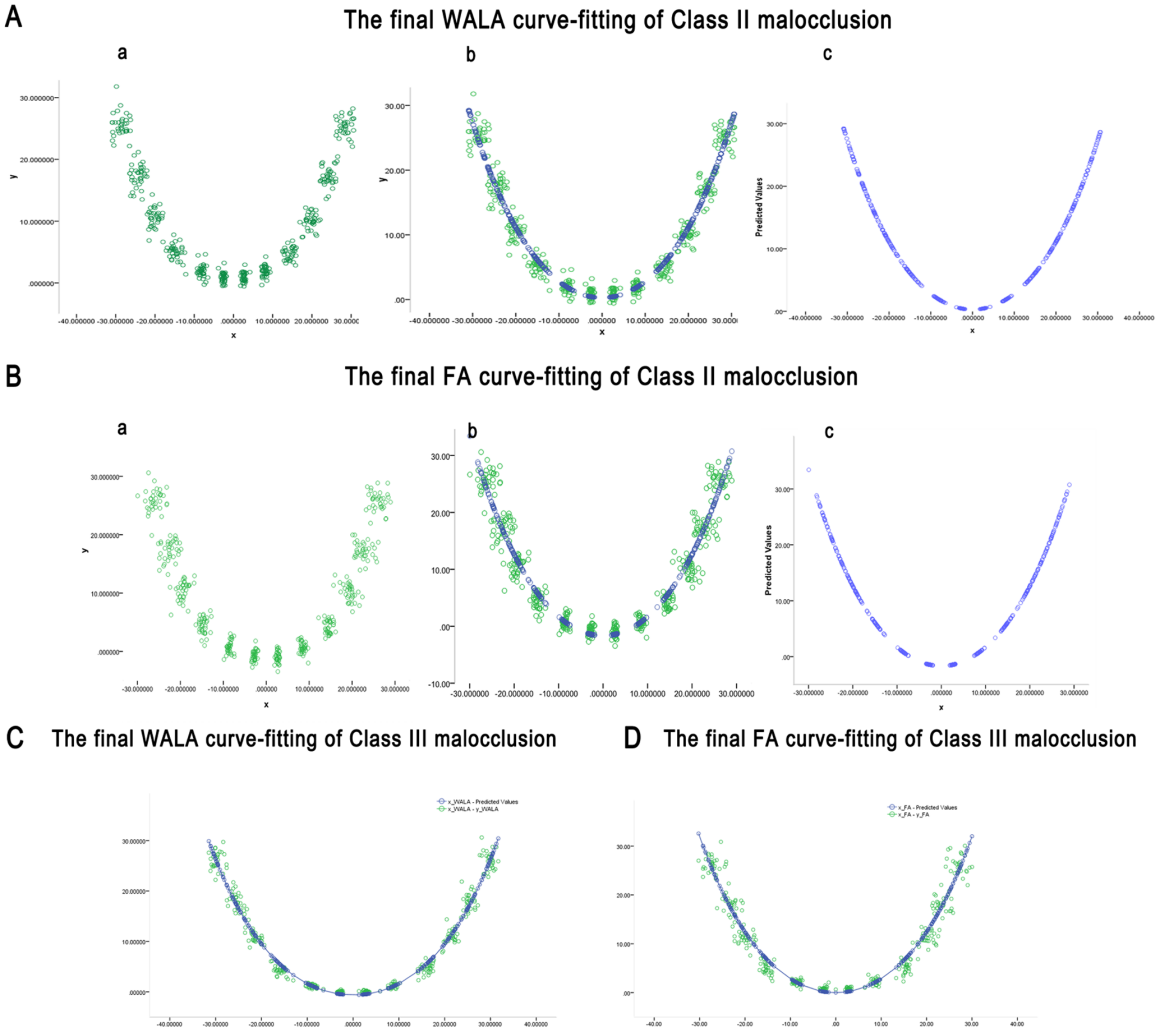
A. The final WALA curve-fitting of Class II malocclusion: a. WALA scatter diagrams of SC2 samples were processed by nonlinear regression analysis via SPSS 17.0. b. The fourth degree even order power function was utilized to analyze the curve fitting to represent the basal bone archform curves. c. The final fitting curve of WALA and the curve equation for WALA is derived: 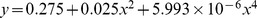. **B. The final FA curve-fitting of Class II malocclusion:** a. FA scatter diagrams of SC2 samples were processed by nonlinear regression analysis via SPSS 17.0. b. The fourth degree even order power function was utilized to analyze the curve fitting to represent the dental archform curves. c. The final fitting curve of FA and the curve equation for FA is derived: 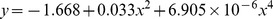. **C. The final WALA curve-fitting of Class III malocclusion.** The curve equation for WALA is derived: 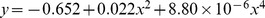. **D. The final FA curve-fitting of Class III malocclusion.** The curve equation for FA is derived: 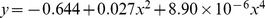.

FA scatter diagrams (fig. 1.B.a) of SC2 samples were processed by nonlinear regression analysis via SPSS 17.0. A fourth degree, even order power function was utilized to analyze curve fitting to represent the dental and basal bone archform curves (fig. 1.B.b, 1.B.c). WALA scatter diagrams and relevant fitting curves of SC2 malocclusions are formed in the same way as the FA curves (fig. 1.A). The curvature radius (r, which reflects the arc line at the anterior area) of the FA fitting curve of the SC2 group is 15.194 (*r_FA2_*), and the regression coefficient (R^2^) is 0.912. The corresponding curve equation is
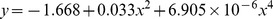



A similar curve equation for WALA is also derived: 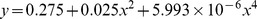



The value of *r_WALA2_* was 20.250, and R^2^ = 0.947. SC3 group curve fitting was accomplished the same way (fig. 1.C, 1.D). The basal curve fitting equation was 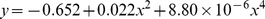
and data show that *r_WALA3_* = 22.47, *R* ^2^ = 0.963.

Similarly, the same FA curve-fitting analysis for SC3 malocclusion demonstrates that *r_FA3_* = 18.18, *R* ^2^ = 0.930 and the fitting equation was 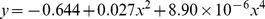



**Figure 2:**
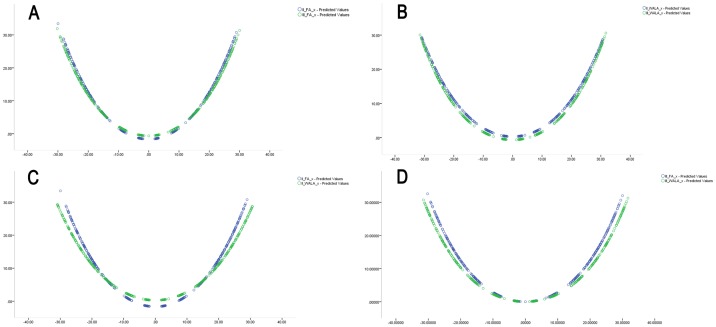
A. The FA curves' comparison between skeletal Class II and III. B. The WALA curves' comparison between skeletal Class II and III. C. The FA and WALA fitting curves of SC2 group. D. The FA and WALA fitting curves of SC3 group.

Data show that the dental arch curve radius of curvature (r) for both groups (fig. 2) was smaller than that of the basal bone curve (*r_WALA3_*>*r_WALA2_*>*r_FA3_*>*r_FA2_*).

## Discussion

### The relative spatial location between the FA and WALA points

In 1972, Andrews suggested the keys to normal occlusion and introduced the concept of inclination [12]. Since that time, clinicians have been studying tooth inclination at posterior sites to explore mechanism of dental and basal transverse discrepancies [15]–[17]. Most of the work has focused on dental archforms of Class II malocclusions, comparing them to normal occlusion or Class I malocclusions. A cross-sectional study by Sayin of 30 Class II division 1 female cases suggested that arch widths were narrower in Class II, division 1 groups, suggesting the transverse discrepancy compensation with the lingual inclination of maxillary molars and the buccal inclination of mandibular molars [10]. Similar findings have been reported by Uysal who compared Class II malocclusions and normal occlusions [18]. In 2013, Rui and colleagues used the modified universal bevel protractor to measure buccalingual teeth inclinations of Class II division 1 malocclusions and Class I occlusions [16]. The reported that buccalingual inclination was vital to the transverse discrepancy of the Class II division 1 group, a finding consistent with the compensation hypothesis of Staley. Although this study did not use measurements of the buccalingual inclination for each tooth, the relative spatial location between FA and WALA points was represented by the distance between the FA and WALA projection's points, respectively. In our work, the inclination's values of the SC3 group are positive, suggesting that mandibular teeth are lingually inclined for the compensation of excessive mandible development. For SC2 malocclusion patients, there were negative mean distances between FA and WALA points at the incisor sites and positive mean values for posterior teeth areas. Also, the degree of lingual inclination at the molar sites of the SC2 group was greater than that of the SC3 malocclusion group. These results suggest that the dental compensation for maxillary deficiencies of SC2 is the bucal position of the maxillary teeth (at incisor sites, teeth buccally positioned to decrease overjet and overbite; at molar sites, teeth less lingually inclined to prevent posterior scissor bite).

### Correlation comparison between dental and basal archforms

Many researchers seek relatively simplified and regular archforms to improve their clinical practice [11], [19], [20]. Some researchers have suggested that basal bone archforms can be used to predetermine dental archforms. Ball [6] compared the relationship between dental and basal archforms in Class II Division 1 and Class I patients, and a relationship was observed between FA and corresponding WALA points in canine and molar areas (R^2^ = 0.843, 0.847). However, Kim [7] reported a moderately relevant relationship in dental and basal archforms in the canine region for normal occlusion samples (r = 0.48), and these results agreed with our findings.

The present study extended this research to SC2 patients, suggesting a moderate correlation between FA and WALA points in the canine region (r = 0.534) and a high correlation in the molar region (r = 0.873). Thus, we could use posterior basal bone archforms and widths to infer the dental archform. Nevertheless, for moderately relevant canine areas, the reference value for making dental archwires may be limited. A similar trend was found in SC3 patients in our study. In addition, the correlation coefficient of the corresponding area was larger in the SC3 group than the SC2 group, revealing that correlation between the dental and basal archform was higher with Class III malocclusions. According to our findings, WALA points were better for predicting individual dental archforms in SC3 malocclusion.

### Comparisons of widths of intercanine and intermolar dimensions

The transverse discrepancy is of interest to many orthodontists. Staley and colleagues reported that the subjects with normal occlusions had larger mandibular dental arch widths than those with Class II division 1 malocclusions, and a significant difference existed in mandibular dental arch widths between the 2 groups [10], [21]. However, Frohlich and Rui suggested that the mandibular dental arch widths were smaller in Class II division 1 patients compared with normally occluded patients or those with Class I malocclusion arches, but there was no significant difference in the widths between these groups [16], [22]. Interestingly, few studies exist to describe dental and basal arches of SC3 malocclusions. In 2010, Slaj considered that Class III patients had larger dental arch widths and depths compared to patients with Class I and II malocclusions [20]. Similarly, Suk and co-workers' research compared dental archforms between SC3 malocclusions and normal occlusions using cone-beam computed tomography [23]. They reported that SC3 subjects had larger intercanine and intermolar dental arch widths compared with patients with normal occlusions, data consistent with our research findings. We found significantly larger intercanine and intermolar widths in the SC3 group when compared to SC2 malocclusion cases.

### Best fit curves of dental and basal bone arch

Many researchers prefer to fit dental arch curves using mathematical functions [24], and the ellipse [25], parabola [26], conic or cubic polynomial [27], [28], fourth-degree polynomial function [29] and the β function [30] have been applied to dental arch curve fitting. Recently, Kim and Kyung used Matlab software to analyze coordinate data to generate a best-fit curve representing the arch by utilizing fourth degree polynomial equations [23], [31]. In our study, data were symmetrical, so an even-power polynomial curve fitting equation was best for smoothing pre-processed data. The fourth degree, even order power function was the best curve for data fitting for synthetic considerations of correlation coefficients and morphologic processes of curve fitting.

According to our results, a narrower intercanine dental arch width in SC2 malocclusion may cause a smaller radius of the dental arch curvature compared with basal arch curvatures. This may explain part of the buccal inclinations of the lower incisors that are clinically observed to accompany deep overbite and deep overjet. In contrast, our data reflects that at the site of the lower incisor, the WALA curve is more flat than the FA curve for the SC3 group. Thus, a more lingual position of the teeth compared to the corresponding location of the basal bone make the dental compensation for the relatively excessive mandible development in SC3 patients.

## Conclusions

Both curves (FA and WALA) representing the archforms were highly individualized. Mandibular dental and basal intercanine and intermolar widths are significantly different between Skeletal Class II and Skeletal Class III groups. Compared with skeletal Class II group, the mandibular arch widths are larger for skeletal Class III group, suggesting that the mandibular arch form were much more smooth for skeletal Class III subjects.

In both groups, mandibular dental and basal widths were moderately correlated in the canine area but were highly correlated in the molar area. Moreover, the correlation coefficient of skeletal Class III is greater than that of skeletal Class II group, which may indicate that it is more appropriate for clinicians to make archwire based on the basal bone archform for skeletal Class III.

Finally, fourth degree, even order power functions were reliable methods for FA and WALA curve-fitting. The fitting curves for each group have obvious differences in morphology.

## Supporting Information

Figure S1 **Locate the FA and WALA points by using the software Rapidform 2006.** FA points: the most prominent part of clinic crown center; for the first molar, it is identified as the most prominent point near the line with the mesiobuccal groove; WALA points: the most prominent point on the soft-tissue ridge immediately superior to the mucogingival junction.
(TIF)

Figure S2 **Establish the 3-D coordinates.** The points “FW1_ref, FW6_ref, and FW6” were chosen to build the 3-D coordinates, and the point “FW1_ref” is the original point with the coordinate (0, 0, 0).
(TIF)

Figure S3 **The average relative distances between corresponding FA and WALA projection points.** The distance between the FA_ref and WALA_ref points for each tooth from right first molar to left first molar was recorded and defined the criterion of positive/negative value; If WALA point is located buccally, the value is positive, otherwise the value is negative;In skeletal Class II group: a negative distance in anterior area and a positive value in posterior area; In skeletal Class III group:positive values in both areas.
(TIF)

Figure S4 **The superimposed curves for patients in both groups.** There are four superimposed curves by connecting the FA and WALA projection points in sequence for both malocclusion groups.
(TIF)

Table S1 **Power Analysis.**
(DOCX)

Table S2 **The STROBE checklist.**
(DOCX)

## References

[1] Handelman CS (1996) The anterior alveolus: its importance in limiting orthodontic treatment and its influence on the occurrence of iatrogenic sequelae. Angle Orthod 66: 95–109 discussion 109–110. 8712499 10.1043/0003-3219(1996)066<0095:TAAIII>2.3.CO;2

[2] Evangelista K, Vasconcelos KdF, Bumann A, Hirsch E, Nitka M, et al (2010) Dehiscence and fenestration in patients with Class I and Class II Division 1 malocclusion assessed with cone-beam computed tomography. American Journal of Orthodontics and Dentofacial Orthopedics 138: 133 –133 20691344 10.1016/j.ajodo.2010.02.021

[3] Lundström AF (1925) Malocclusion of the teeth regarded as a problem in connection with the apical base. International Journal of Orthodontia, Oral Surgery and Radiography 11: 1022–1042.

[4] Howes A (1954) A polygon portrayal of coronal and basal arch dimendions in the horizontal plane. Am J Orthod Dentofacial Orthop 40: 811–831.

[5] Andrews LF, Andrews W (2000) The six elements of orificial harmony. Andrews J 1: 13–22.

[6] Ball RL, Miner RM, Will LA, Arai K (2010) Comparison of dental and apical base arch forms in Class II Division 1 and Class I malocclusions. Am J Orthod Dentofacial Orthop 138: 41–50. 20620832 10.1016/j.ajodo.2008.11.026

[7] Kim K-Y, Bayome M, Kim K, Han SH, Kim Y, et al (2011) Three-dimensional evaluation of the relationship between dental and basal arch forms in normal occlusion. Korean Journal of Orthodontics 41: 288–296.

[8] Ronay V, Miner RM, Will LA, Arai K (2008) Mandibular arch form: The relationship between dental and basal anatomy. Am J Orthod Dentofacial Orthop 134: 430–438. 18774089 10.1016/j.ajodo.2006.10.040

[9] Lux CJ, Conradt C, Burden D, Komposch G (2003) Dental arch widths and mandibular-maxillary base widths in Class II malocclusions between early mixed and permanent dentitions. Angle Orthod 73: 674–685. 14719732 10.1043/0003-3219(2003)073<0674:DAWAMB>2.0.CO;2

[10] Sayin MO, Turkkahraman H (2004) Comparison of dental arch and alveolar widths of patients with Class II, division 1 malocclusion and subjects with Class I ideal occlusion. Angle Orthod 74: 356–360. 15264647 10.1043/0003-3219(2004)074<0356:CODAAA>2.0.CO;2

[11] Triviño T, Siqueira DF, Scanavini MA (2008) A new concept of mandibular dental arch forms with normal occlusion. American Journal of Orthodontics and Dentofacial Orthopedics 133: 10.e15–10.e22. 10.1016/j.ajodo.2007.07.014 18174064

[12] Andrews LF (1972) The six keys to normal occlusion. Am J Orthod Dentofacial Orthop 62: 296–309. 10.1016/s0002-9416(72)90268-0 4505873

[13] Fleiss (1986) The design and analysis of clinical expriments. New York, NY: Wiley.

[14] Kirdwood BR, Sterne JA (2006) Essential medical statistics. 2nd ed. Boston, MA: Blackwell Science.

[15] Shewinvanakitkul W, Hans MG, Narendran S, Palomo JM (2011) Measuring buccolingual inclination of mandibular canines and first molars using CBCT. Orthod Craniofac Res 14: 168–174. 21771272 10.1111/j.1601-6343.2011.01518.x

[16] Shu R, Han X, Wang Y, Xu H, Ai D, et al (2013) Comparison of arch width, alveolar width and buccolingual inclination of teeth between Class II division 1 malocclusion and Class I occlusion. The Angle orthodontist 83: 246–252. 23458279 10.2319/052412-427.2 PMC8793640

[17] Zachrisson, Bjorn U (2003) Premolar extraction and smile esthetics. Am J Orthod Dentofacial Orthop 124: 11A–12A. 10.1016/j.ajodo.2003.10.005 14680017

[18] Uysal T, Memili B, Usumez S, Sari Z (2005) Dental and alveolar arch widths in normal occlusion, class II division 1 and class II division 2. Angle Orthod 75: 941–947. 16448235 10.1043/0003-3219(2005)75[941:DAAAWI]2.0.CO;2

[19] Lee SJ, Lee S, Lim J, Park HJ, Wheeler TT (2011) Method to classify dental arch forms. Am J Orthod Dentofacial Orthop 140: 87–96. 21724092 10.1016/j.ajodo.2011.03.016

[20] Slaj M, Spalj S, Pavlin D, Illes D (2010) Dental archforms in dentoalveolar Class I, II and III. Angle Orthod 80: 919–924. 20578864 10.2319/112609-672.1 PMC8939017

[21] Staley RN, Stuntz WR, Peterson LC (1985) A comparison of arch widths in adults with normal occlusion and adults with class II, Division 1 malocclusion. Am J Orthod 88: 163–169. 3861102 10.1016/0002-9416(85)90241-6

[22] Frohlich F (1961) A longitudinal study of untreated Class II type malocclusion. Trans Eur Orthop Soc 37: 137–139.

[23] Suk KE, Park JH, Bayome M, Nam YO, Sameshima GT, et al (2013) Comparison between dental and basal arch forms in normal occlusion and Class III malocclusions utilizing cone-beam computed tomography. Korean J Orthod 43: 15–22. 23504406 10.4041/kjod.2013.43.1.15 PMC3594875

[24] Pepe SH (1975) Polynomial and catenary curve fits to human dental arches. J Dent Res 54: 1124–1132. 1059650 10.1177/00220345750540060501

[25] Currier JH (1969) A computerized geometric analysis of human dental arch form. Am J Orthod 56: 164–179. 4896226 10.1016/0002-9416(69)90232-2

[26] Jones ML, Richmond S (1989) An assessment of the fit of a parabolic curve to pre- and post-treatment dental arches. Br J Orthod 16: 85–93. 2673344 10.1179/bjo.16.2.85

[27] Sampson P (1981) Dental arch shape:A statistical analysis using conic sections. Am J Orthod Dentofacial Orthop 79: 535–547. 10.1016/s0002-9416(81)90464-4 6940448

[28] BeGole EA (1980) Application of the cubic spline function in the description of dental arch form. J Dent Res 59: 1549–1556. 6931142 10.1177/00220345800590092901

[29] Sanin C, Savara BS, Thomas DR, Clarkson QD (1970) Arc length of the dental arch estimated by multiple regression. J Dent Res 49: 885. 5269390 10.1177/00220345700490042801

[30] Braun S, Hnat WP, Fender DE, Legan HL (1998) The form of the human dental arch. Angle Orthod 68: 29–36. 9503132 10.1043/0003-3219(1998)068<0029:TFOTHD>2.3.CO;2

[31] Kim JT, Lee JW (2011) Three dimensional structural analysis between dental arch and basal bone in normal occlusion. Korean J Orthod 41: 224–236.

