# Supplementary figures and images for: Archform Comparisons between Skeletal Class II and III Malocclusions
Source: PLoS One. 2014 Jun 27;9(6):e100655. doi: 10.1371/journal.pone.0100655 (PMC4074102; doi:10.1371/journal.pone.0100655)

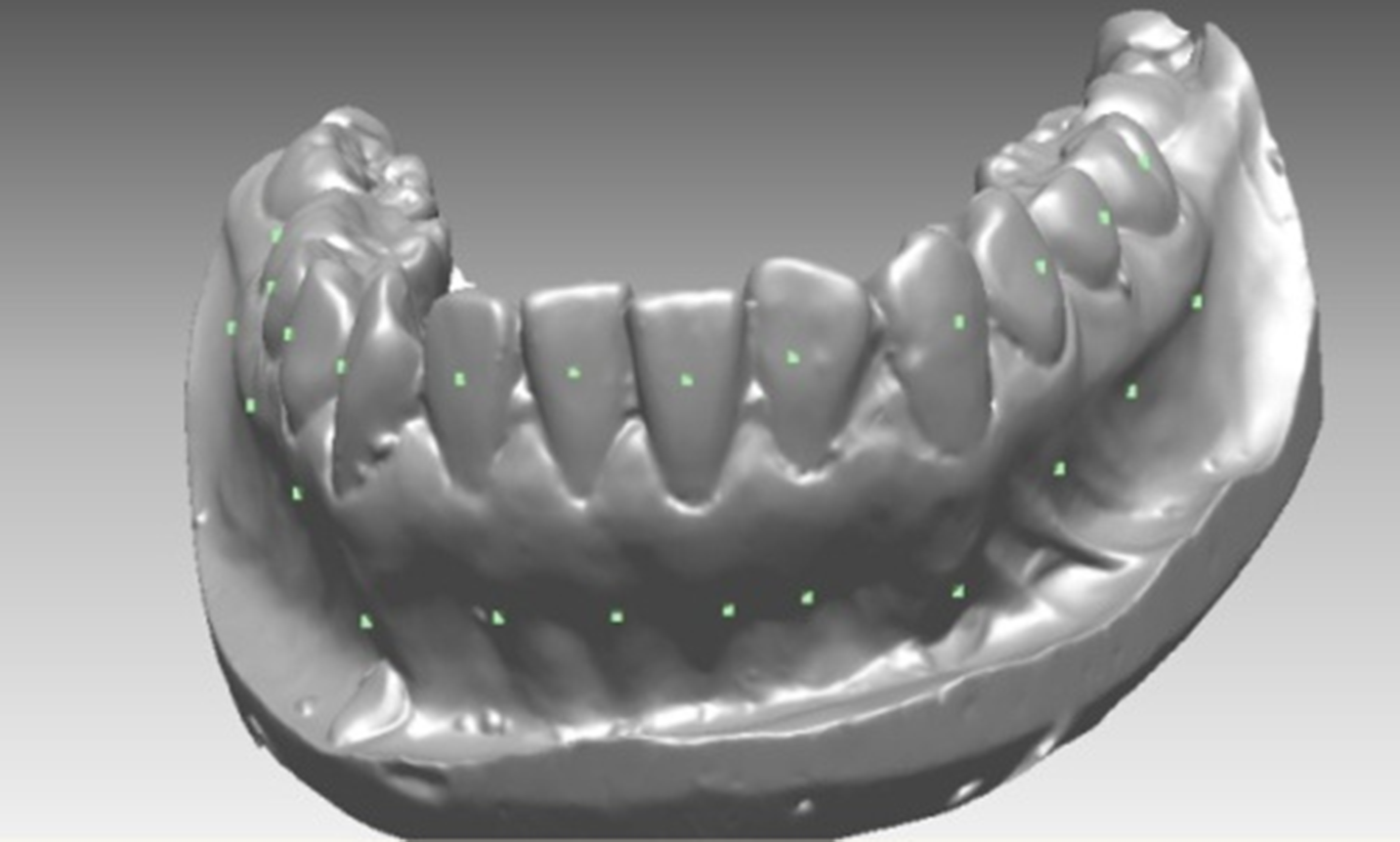

Supplement: Figure S1 — Locate the FA and WALA points by using the software Rapidform 2006. FA points: the most prominent part of clinic crown center; for the first molar, it is identified as the most prominent point near the line with the mesiobuccal groove; WALA points: the most prominent point on the soft-tissue ridge immediately superior to the mucogingival junction. (TIF) [file pone.0100655.s001.tif]

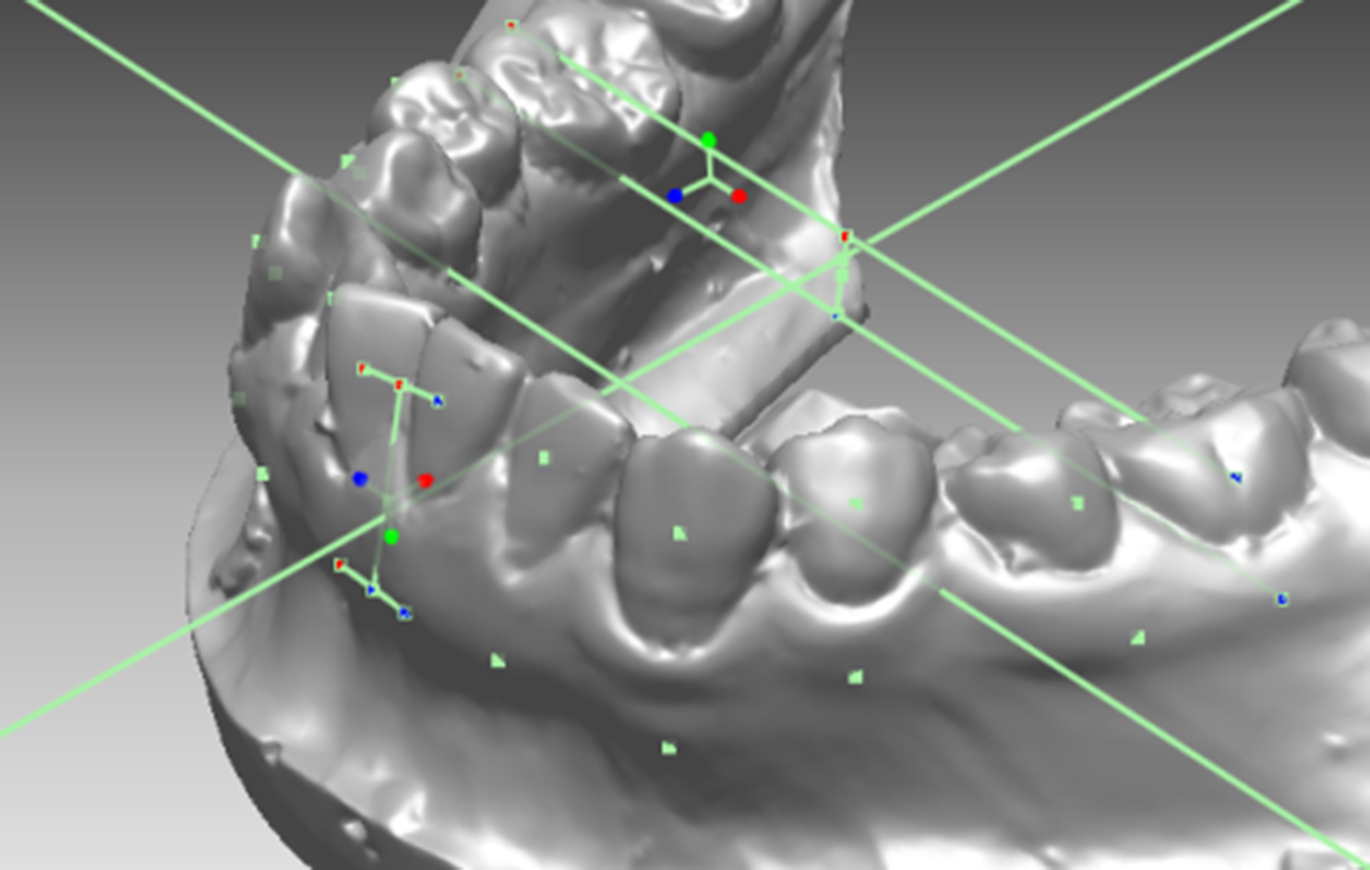

Supplement: Figure S2 — Establish the 3-D coordinates. The points “FW1_ref, FW6_ref, and FW6” were chosen to build the 3-D coordinates, and the point “FW1_ref” is the original point with the coordinate (0, 0, 0). (TIF) [file pone.0100655.s002.tif]

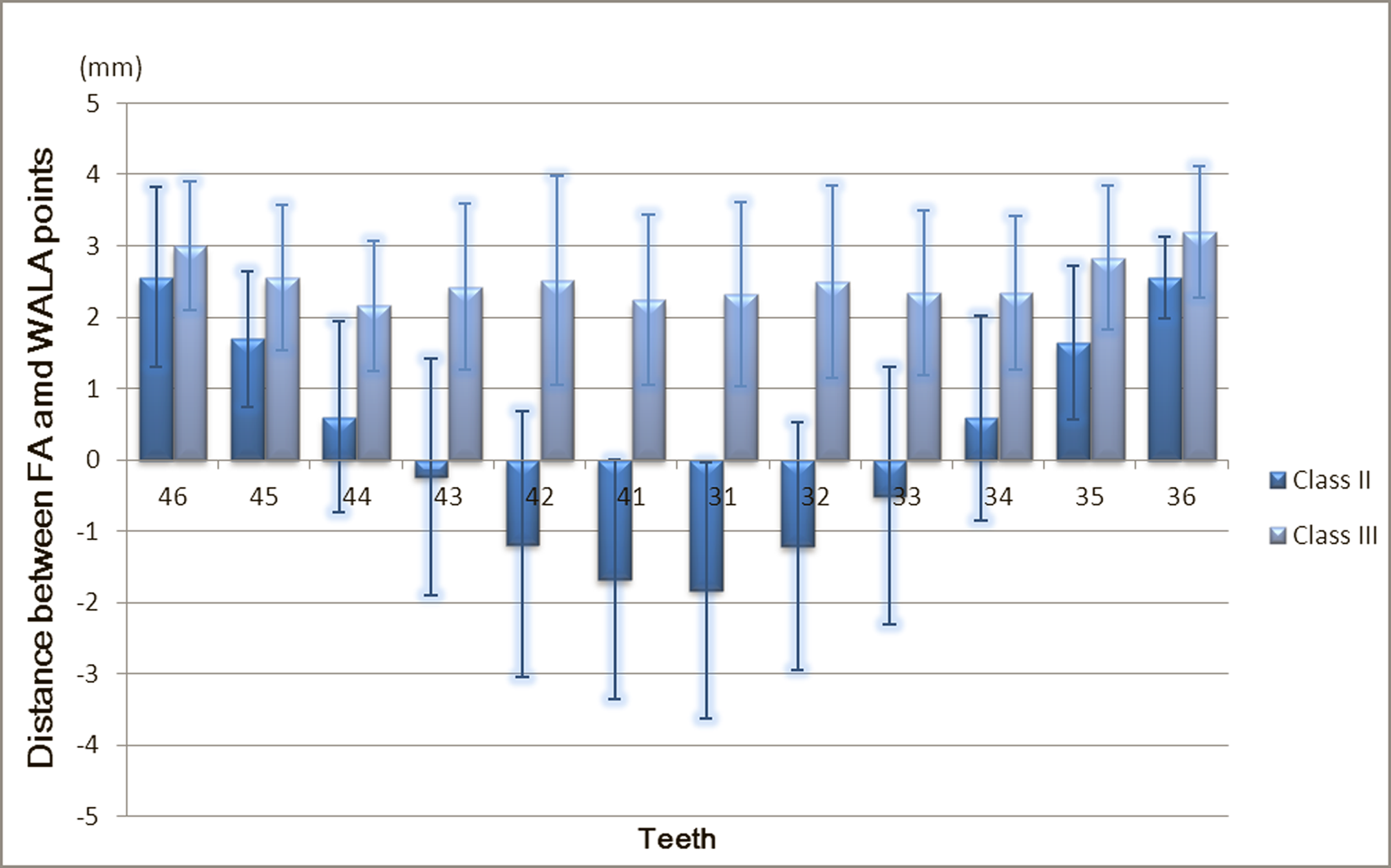

Supplement: Figure S3 — The average relative distances between corresponding FA and WALA projection points. The distance between the FA_ref and WALA_ref points for each tooth from right first molar to left first molar was recorded and defined the criterion of positive/negative value; If WALA point is located buccally, the value is positive, otherwise the value is negative;In skeletal Class II group: a negative distance in anterior area and a positive value in posterior area; In skeletal Class III group:positive values in both areas. (TIF) [file pone.0100655.s003.tif]

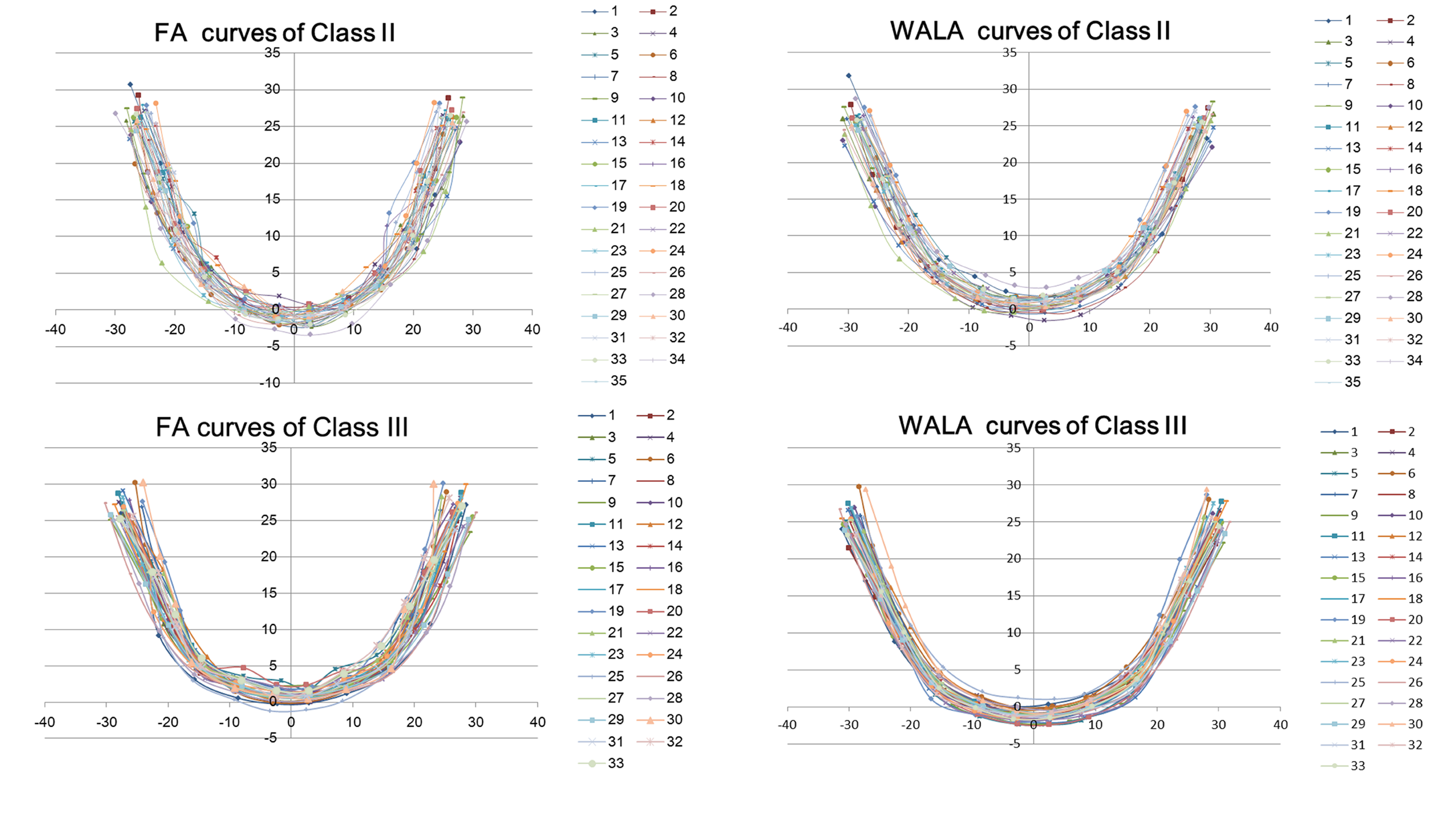

Supplement: Figure S4 — The superimposed curves for patients in both groups. There are four superimposed curves by connecting the FA and WALA projection points in sequence for both malocclusion groups. (TIF) [file pone.0100655.s004.tif]
