# Supplementary material for: Archform Comparisons between Skeletal Class II and III Malocclusions
Source: PLoS One. 2014 Jun 27;9(6):e100655. doi: 10.1371/journal.pone.0100655 (PMC4074102; doi:10.1371/journal.pone.0100655)
Supplement: Table S1 — Power Analysis. (DOCX) [file pone.0100655.s005.docx]

**Table S1：Power Analysis**

| t tests - Means: Difference between two independent means (two groups) | | | |
| --- | --- | --- | --- |
| Analysis: | A priori: Compute required sample size | | |
| Input: | Tail(s) | = | Two |
|  | Effect size d | = | 0.6859943 |
|  | α err prob | = | 0.05 |
|  | Power (1-β err prob) | = | 0.8 |
|  | Allocation ratio N2/N1 | = | 1 |
| Output: | Noncentrality parameter δ | = | 2.86972 |
|  | Critical t | = | 1.9954689 |
|  | Df | = | 68 |
|  | Sample size group 1 | = | 35 |
|  | Sample size group 2 | = | 35 |
|  | Total sample size | = | 70 |
|  | Actual power | = | 0.8075793 |
