# Supplementary material for: Archform Comparisons between Skeletal Class II and III Malocclusions
Source: PLoS One. 2014 Jun 27;9(6):e100655. doi: 10.1371/journal.pone.0100655 (PMC4074102; doi:10.1371/journal.pone.0100655)
Supplement: Table S2 — The STROBE checklist. (DOCX) [file pone.0100655.s006.docx]

**The STROBE checklist**

Archform Comparisons between Skeletal Class II and III Malocclusions

| Item Name | Item number | Cross-sectional study |
| --- | --- | --- |
| TITLE and ABSTRACT | 1 | We have indicated that our study is a cross-sectional research in the part of the abstract. |
| *INTRODUCTION* | | |
| BACKGROUND/ RATIONALE | 2 | Dental and basal bone archforms for normal occlusions have been studied as have those for Class I and Class II malocclusions. However, the association between dental and basal bone arches for Skeletal Class III counterparts is unclear. |
| OBJECTIVE | 3 | The purpose of this research was to explore the relationship of the mandibular dental and basal bone archforms between severe Skeletal Class II and Skeletal Class III malocclusions. |
| *METHOD* | | |
| STUDY DESIGN | 4 | We compared the mandibular archforms between skeletal Class II and III malocclusions by 3-D models analysis. |
| SETTING | 5 | The samples were comprised of 68 mandibular pretreatment casts obtained from subjects with 35 skeletal Class II and 33 skeletal Class III malocclusions from the year of 2010 to 2013. |
| PARTICIPANTS | 6 | Inclusion criterions were as follows:  1) All subjects are pretreatment natural models (no distortions; clear teeth surface).  2) The treatment plans are confirmed as combined orthognathic and orthodontic treatments.  3) Minor arch length discrepancy for the lower dental arch (crowding ≤ 2 mm，spacing ≤ 2 mm).  4) For skeletal Class II: A canine and molar Class II relationship and ANB angle >5°  5) For skeletal Class III: A canine and molar Class III relationship and ANB angle <-5° |
| VARIABLES | 7 | **Outcome**：1. average relative distances between corresponding FA and WALA projection points；2. intercanine and intermolar widths for FA and WALA points; 3. Curve fitting of FA and WALA’s projection points.  **Exposures**：skeletal discrepancy  **Potential confounders**：the inaccuracy of digitizing the FA and WALA points from different times and different person. |
| DATA SOURCE MEASUREMENT | 8 | **Outcome**：1. average relative distances between corresponding FA and WALA projection points；2. intercanine and intermolar widths for FA and WALA points; 3. Curve fitting of FA and WALA’s projection points: A fourth degree, even order power function was utilized to analyze curve fitting to represent the dental and basal bone archform curves. The values of curvature radius were compared between two groups.  **Exposures**：skeletal discrepancy  **Potential confounders**：the inaccuracy of digitizing the FA and WALA points from different times and different person. |
| BIAS | 9 | To evaluate intra-operator reliability of FA and WALA point identification, all selected models were re-digitized 2 weeks later by the same operators. The intraclass correlation coefficient (ICC>0.8) indicated high reliability for point locations and archform measurements. Data averages were used as final measurement outcomes in the data processing and statistical analysis. |
| STUDY SIZE | 10 | We use the G-power software to calculate the estimated study size (each group for 35 samples) and the process are presented as followed:  t tests - Means: Difference between two independent means (two groups)  Analysis: A priori: Compute required sample size  Input: Tail(s) = Two  Effect size d = 0.6859943  α err prob = 0.05  Power (1-β err prob) = 0.8  Allocation ratio N2/N1 = 1  Output: Noncentrality parameter δ = 2.8697200  Critical t = 1.9954689  Df = 68  Sample size group 1 = 35  Sample size group 2 = 35  Total sample size = 70  Actual power = 0.8075793 |
| QUANTITATIVE VARIABLES | 11 | 1．The distances between FA and WALA points for each tooth；2. intercanine and intermolar widths for FA and WALA points;  3. The curvature radius of the FA and WALA fitting curve of 2 groups. |
| STATISTICAL METHODS | 12 | 1. The data were imported into SPSS 17.0 and subjected to the Kolmogorov-Smirnov test to analyze normality. 2. Data from intercanine widths with abnormal distributions, and dental and basal bone widths in the canine region were compared using the rank-sum test. Otherwise, data were evaluated with the independent samples t-test. 3. Correlation statistics were used to assess the correlation between the FA and WALA point distances at the canine and molar areas. 4. Pearson correlation coefficients between the intercanine and intermolar widths at the FA and WALA points were also calculated and compared between 2 groups. 5. Scatter diagrams for samples were processed by nonlinear regression analysis. |
| *RESULTS* | | |
| PARTICIPANTS | 13 | Skeletal Class II：35 patients  Skeletal Class III：33 patients |
| DESCRIPTIVE DATA | 14 | **Skeletal Class II (SC2)：**  A canine and molar Class II relationship and ANB angle >5°  **Skeletal Class III(SC3)：**  A canine and molar Class III relationship and ANB angle <-5° |
| OUTCOME DATA | 15 | 1.The mandibular dental and basal bone intercanine and intermolar widths were significantly greater in the SC3 group compared to the SC2 group.  2. In both groups, a moderate correlation existed between dental and basal bone arch widths in the canine region, and a high correlation existed between dental and basal bone arch widths in the molar region.  3. The coefficient of correlation of the SC3 group was greater than the SC2 group.  4．Fourth degree, even order power functions were used as best-fit functions to fit the scatter plots.  5．The radius of curvature was larger in SC3 malocclusions compared to SC2 malocclusions (*rWALA3＞rWALA2＞rFA3＞rFA2*). |
| MAIN RESULTS | 16 | The same with the item 15 |
| OTHER ANALYSES | 17 | None |
| *DISCUSSION* | | |
| KEY RESULTS | 18 | Mandibular dental and basal intercanine and intermolar widths were significantly different between the two groups. Compared with SC2 subjects, the mandibular archform was more flat for SC3 subjects. |
| LIMITATION | 19 | Our research samples were chosen from Mongolian ancestry, therefore，the results can not represent all races. |
| INTERPRETATION | 20 | 1. A cross-sectional study by Sayin of 30 Class II division 1 female cases suggested that arch widths were narrower in Class II, division 1 groups, suggesting a transverse discrepancy compensation with the lingual inclination of maxillary molars and the buccal inclination of mandibular molars (Sayin and Turkkahraman, 2004). Similar findings have been reported by Uysal who compared Class II malocclusions and normal occlusions (Uysal et al., 2005). In 2013, Rui and colleagues used the modified universal bevel protractor to measure buccalingual teeth inclinations of Class II division 1 malocclusions and Class I occlusions (Shu et al., 2013). The reported that buccalingual inclination was vital to the transverse discrepancy of the Class II division 1 group, a finding consistent with the compensation hypothesis of Staley. In our work, the inclination’s values of the SC3 group are positive, suggesting that mandibular teeth are lingually inclined for the compensation of excessive mandible development. 2. Some researchers have suggested that basal bone archforms can be used to predetermine dental archforms. Ball (Ball et al., 2010) compared the relationship between dental and basal archforms in Class II Division 1 and Class I patients, and a relationship was observed between FA and corresponding WALA points in canine and molar areas. However, Kim (Kim et al., 2011) reported a moderately relevant relationship in dental and basal archforms in the canine region for normal occlusion samples, and these results agreed with our findings. The present study extended this research to SC2 patients, suggesting a moderate correlation between FA and WALA points in the canine region and a high correlation in the molar region. 3. Staley and colleagues reported that the subjects with normal occlusions had larger mandibular dental arch widths than those with Class II division 1 malocclusions, and a significant difference existed in mandibular dental arch widths between the 2 groups. However, Frohlich and Rui suggested that the mandibular dental arch widths were smaller in Class II division 1 patients compared with normally occluded patients or those with Class I malocclusion arches, but there was no significant difference in the widths between these groups (Frohlich, 1961; Shu et al., 2013). In 2010, Slaj considered that Class III patients had larger dental arch widths and depths compared to patients with Class I and II malocclusions (Slaj et al., 2010). Similarly, Suk and co-workers’ research compared dental archforms between SC3 malocclusions and normal occlusions using cone-beam computed tomography (Suk et al., 2013). We found significantly larger intercanine and intermolar widths in the SC3 group when compared to SC2 malocclusion cases. 4. Many researchers prefer to fit dental arch curves using mathematical functions (Pepe, 1975), and the ellipse (Currier, 1969), parabola (Jones and Richmond, 1989), conic or cubic polynomial (Sampson, 1981; BeGole, 1980), fourth-degree polynomial function (Sanin et al, 1970) and the β function (Braun et al, 1998) have been applied to dental arch curve fitting. Kim and Kyung used Matlab software to analyze coordinate data to generate a best-fit curve representing the arch by utilizing fourth degree polynomial equations (Kim and Lee, 2011; Suk et al., 2013). In our study, data were symmetrical, so an even-power polynomial curve fitting equation was best for smoothing pre-processed data. The fourth degree, even order power function was the best curve for data fitting for synthetic considerations of correlation coefficients and morphologic processes of curve fitting. |
| GENERALIZABILITY | 21 | 1.Mandibular dental and basal intercanine and intermolar widths are significantly different between SC2 and SC3 groups and in both groups;  2.Mandibular dental and basal widths were moderately correlated in the canine area but were highly correlated in the molar area.  3.Fourth degree, even order power functions were reliable methods for FA and WALA curve-fitting. |
| *OTHER INFORMATION* | | |
| FUNDING | 22 | the National Natural Science Foundation of China （grant numbers 81171006）; the Beijing Science and Technology Committee （grant number Z121107001012024） |
